# Global research trends of *Mycoplasma pneumoniae* pneumonia in children: a bibliometric analysis

**DOI:** 10.3389/fped.2023.1306234

**Published:** 2023-11-24

**Authors:** Zhe Song, Guangyuan Jia, Guangzhi Luo, Chengen Han, Baoqing Zhang, Xiao Wang

**Affiliations:** ^1^ The First Clinical Medical College, Shandong University of Traditional Chinese Medicine, Jinan, China; ^2^ Department of Pediatrics, Affiliated Hospital of Shandong University of Traditional Chinese Medicine, Jinan, China; ^3^ College of Traditional Chinese Medicine, Shandong University of Traditional Chinese Medicine, Jinan, China

**Keywords:** *Mycoplasma pneumoniae* pneumonia (MPP), children, bibliometric analysis, research trends, hotspots

## Abstract

**Background:**

*Mycoplasma pneumoniae* pneumonia (MPP), attributable to *Mycoplasma pneumoniae* (MP), represents a predominant form of community-acquired pneumonia in pediatric populations, thereby posing a significant threat to pediatric health. Given the burgeoning volume of research literature associated with pediatric MPP in recent years, it becomes imperative to undertake a bibliometric analysis aimed at delineating the current research landscape and emerging trends, thereby furnishing a framework for subsequent investigations.

**Methods:**

A comprehensive literature search targeting pediatric MPP was conducted in the Web of Science Core Collection. After the removal of duplicate entries through Endnote software, the remaining articles were subject to scientometric analysis via Citespace software, VOSviewer software and R language, focusing on variables such as publication volume, contributing nations, institutions and authors, references and keywords.

**Results:**

A total of 1,729 articles pertinent to pediatric MPP were included in the analysis. China and the United States emerged as the nations with the highest publication output. Italian scholar Susanna Esposito and Japanese scholar Kazunobu Ouchi were the most influential authors in the domain of pediatric MPP. Highly-cited articles primarily focused on the epidemiological investigation of pediatric MPP, the clinical characteristics and treatment of macrolide-resistant MPP, and biomarkers for refractory *Mycoplasma pneumoniae* pneumonia (RMPP). From the corpus of 1,729 articles, 636 keywords were extracted and categorized into ten clusters: Cluster #0 centered on molecular-level typing of macrolide-resistant strains; Cluster #1 focused on lower respiratory tract co-infections; Clusters #2 and #6 emphasized other respiratory ailments caused by MP; Cluster #3 involved biomarkers and treatment of RMPP; Clusters #4 and #9 pertained to extrapulmonary complications of MPP, Clusters #5 and #7 addressed etiological diagnosis of MPP, and Cluster #8 explored pathogenic mechanisms.

**Conclusions:**

The past few years have witnessed extensive attention directed towards pediatric MPP. Research in pediatric MPP principally revolves around diagnostic techniques for MP, macrolide resistance, complications of MPP, treatment and diagnosis of RMPP, and elucidation of pathogenic mechanisms. The present study provides pediatric clinicians and researchers with the research status and focal points in this field, thereby guiding the orientation of future research endeavors.

## 1. Introduction

*Mycoplasma pneumoniae* (MP) is presently recognized as the smallest prokaryotic microorganism capable of independent survival without a host cell (1), and concurrently serves as the leading etiological agent responsible for pediatric community-acquired pneumonia (CAP) (2). Globally, epidemics of *Mycoplasma pneumoniae* pneumonia (MPP) materialize at intervals of 3–7 years, accounting for more than 40% of pediatric CAP cases during epidemic years (3). It was previously believed that MPP usually occured in children over five years old, and the symptoms were mild (4, 5). However, recent clinical observations have indicated a trend towards a younger age of onset for MPP (6), accompanied by a rise in cases of refractory pneumonia and multisystemic complications induced by MP, thereby posing a substantial threat to pediatric health (7).

In light of these factors, there has been heightened attention towards this malady from both pediatric clinicians and scientific researchers, culminating in the prolific dissemination of research findings in the form of academic articles. On one hand, this surge in published literature has significantly propelled advancements in the field of pediatric MPP and fostered collaborative endeavors. On the other hand, due to the voluminous, dispersed, and at times redundant nature of these publications, obtaining a comprehensive and systematic understanding of the current state of research on pediatric MPP remains challenging. Bibliometrics, an interdisciplinary field employing mathematical and statistical methodologies, serves to conduct quantitative analysis of the knowledge carriers within a specific academic domain (8). By executing a bibliometric analysis on the extant literature, one can obtain outstanding contributors and current research hotspots in this field, which is beneficial for researchers to identify partners and design research subject in the future. However, to the best of our knowledge, there has yet to be a bibliometric study specifically targeting pediatric MPP. Consequently, the objective of the present study is to deploy bibliometric techniques to integrate existing theoretical, clinical, and experimental findings, thereby manifesting the research advancements in pediatric MPP through knowledge mapping. The study further aims to analyze the prevailing research hotspots and cutting-edge theories within this domain, with the intention of furnishing a referential framework for future investigations.

## 2. Materials and methods

### 2.1. Data source and retrieval strategy

**Figure 1:**
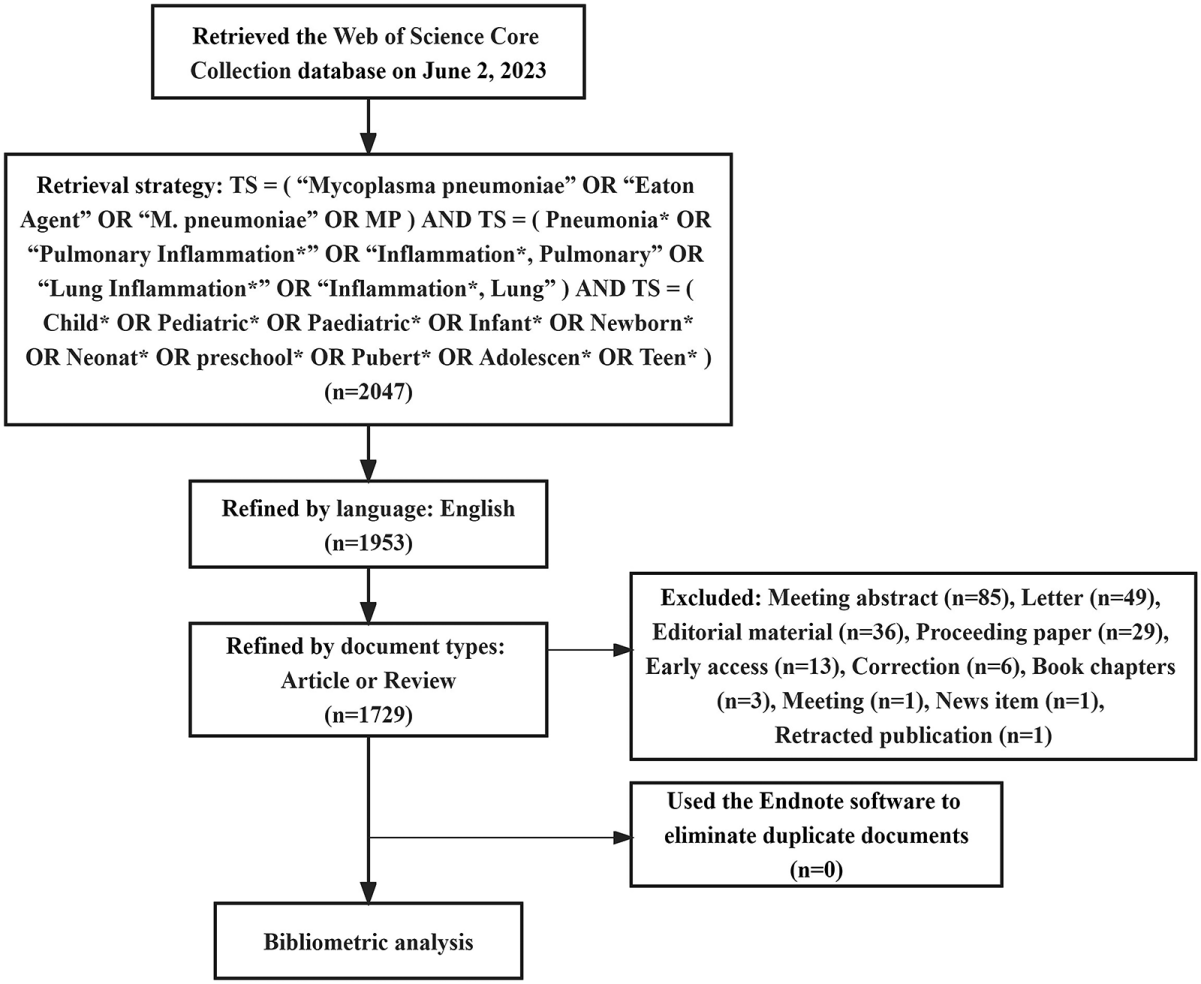
Flow chart of literature search.

The Web of Science is the most commonly used database for bibliometrics research. It contains academic journals, monographs, and conference proceedings in multidisciplinary fields (9). Consequently, this study sourced its corpus of literature from the Web of Science Core Collection (WoSCC). The retrieval strategy employed was articulated as follows: TS = (“*Mycoplasma pneumoniae*” or “Eaton Agent” or “M. pneumoniae” or “MP”) and TS = (Pneumonia* or “Pulmonary Inflammation*” or “Inflammation*, Pulmonary” or “Lung Inflammation*” or “Inflammation*, Lung”) AND TS = (Child* or Pediatric* or Paediatric* or Infant* or Newborn* or Neonat* or Preschool* or Pubert* or Adolescen* or Teen*). Among them, “TS” denotes “Topic Search”; asterisks serve as truncation symbols, capable of representing zero or more succeeding characters (e.g., Child* may signify Child, Children, or Childhood). The time-frame was delimited to include works published up to June 2, 2023. Language criteria were restricted to English, and document type was confined to either “article” or “review”. Duplicate entries were excised utilizing Endnote software (version X9.3.3), culminating in a dataset of 1,729 articles for ensuing analysis. The schematic representation of the literature retrieval procedure is delineated in Figure 1.

### 2.2. Bibliometric analysis

Subsequent to retrieving a total of 1,729 articles from the WoSCC, we downloaded the “Full Record and Cited References” and stored them as plain text files. Predominantly, bibliometric analyses were executed using Citespace software (version 6.2.R3), VOSviewer software (version 1.6.18) and R language's bibliometrix package, focusing on the parameters of publication volume, national contributions, institutional affiliations, authorship, reference co-citations, and keywords.

## 3. Results

### 3.1. Number and growth trend of publications

**Figure 2:**
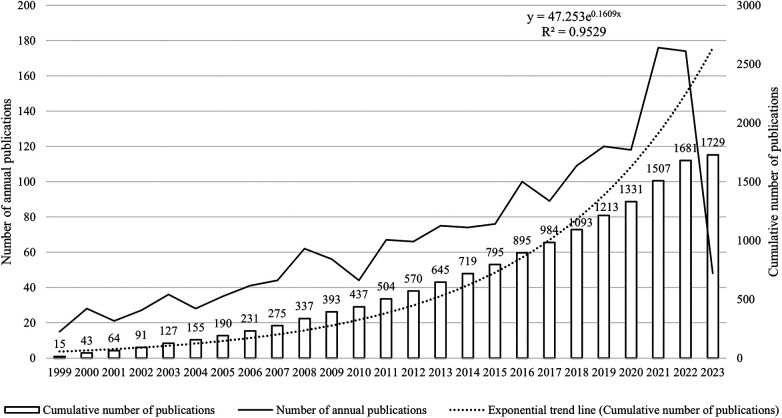
Trend chart of annual number of publications and cumulative number of publications.

In compliance with our retrieval strategy and criteria for inclusion and exclusion, a corpus of 1,729 articles pertinent to pediatric MPP was amassed. Of these, 1,518 were categorized as original research articles, accounting for approximately 90% of the total, while an additional 211 were review articles. As depicted in Figure 2, the cumulative publication volume from 1999 through 2023 approximates an exponential growth pattern (*R*^2^ = 0.9529). Although year-to-year fluctuations in publication counts were observed, the overarching trend manifested a robust increase, notably in the years 2021 and 2022, where the growth was most conspicuous.

### 3.2. Analysis of nations/regions and institutions

**Figure 3:**
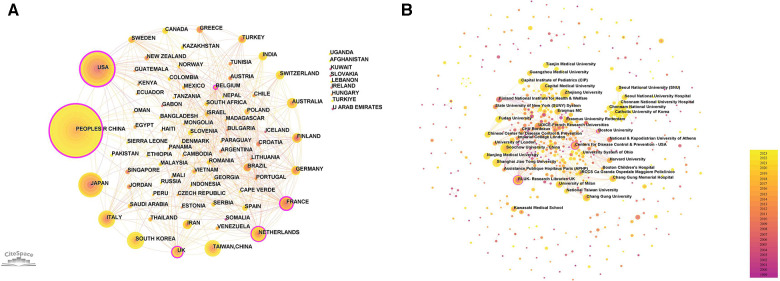
National/regional and institutional cooperation network. (**A**) National/regional cooperation network. Scotland, England and Wales are collectively called UK. New Caledonia is included in France. (**B**) Institutional collaboration network.

Utilizing Citespace software, networks delineating national and institutional collaborations were constructed respectively, as illustrated in Figure 3. Within this graphical representation, each node is emblematic of a nation/region or an institution. The magnitude of the node is indicative of its publication volume, while the color designates the year of publication. Nodes encircled by a purple perimeter possess elevated levels of betweenness centrality (centrality >0.1), signifying their pivotal role in catalyzing international collaborations or institutional communication. As discerned from Figure 3A and Table 1, a total of 86 nations/regions have engaged in pediatric MPP research. China has published 595 articles, constituting 34.4% of the total publication volume and thereby occupying the foremost position. Following China are the United States, Japan, Italy, and South Korea. Notably, the collaborative network among global nations/regions manifests as an intricate web of partnerships. The United States exhibits the highest level of betweenness centrality, indicating its significant contribution to facilitating international exchanges and cooperation.

**Table 1:** Top 5 countries in terms of number of publications and betweenness centrality.

Rank	Country	*N*	Centrality	Rank	Country	Centrality	*N*
1	Peoples R China	595	0.23	1	USA	0.44	292
2	USA	292	0.44	2	France	0.25	52
3	Japan	133	0	3	Peoples R China	0.23	595
4	Italy	96	0.09	4	Netherlands	0.16	56
5	South Korea	83	0	5	UK	0.15	68

Figure 3B and Table 2 display the relevant institutions engaged in pediatric MPP research. Soochow University and Capital Medical University emerged as the institution with the highest publication volume (*n* = 59). Most institutions tend to conduct research independently, and the only institutions with high centrality are the Centers for Disease Control & Prevention in the United States, Boston University and Research Libraries UK.

**Table 2:** Top 5 institutions in terms of number of publications and betweenness centrality.

Rank	Institution	*N*	Centrality	Rank	Institution	Centrality	*N*
1	Soochow University	59	0	1	Centers for Disease Control & Prevention - USA	0.14	26
2	Capital Medical University	59	0.1	2	Boston University	0.13	4
3	Zhejiang University	47	0.01	3	RLUK - Research Libraries UK	0.11	33
4	Shanghai Jiao Tong University	39	0.02	4	Capital Medical University	0.1	59
5	RLUK - Research Libraries UK	33	0.11	5	Chinese Center for Disease Control & Prevention	0.09	21

### 3.3. Analysis of influential authors

**Figure 4:**
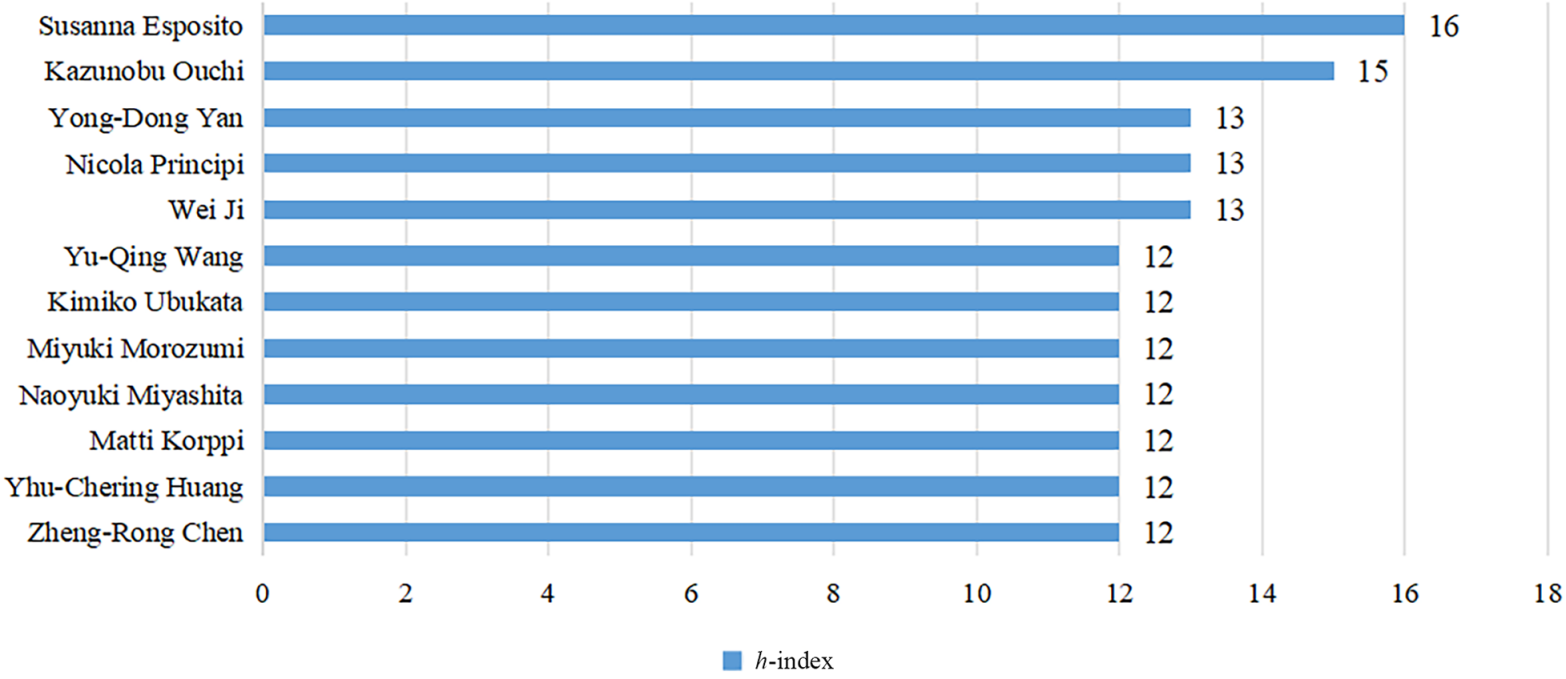
Top 10 authors with *h*-index.

The *h*-index is a commonly used indicator to characterize the scientific output of authors in bibliometrics research (10). It represents that a given author has published *h* papers, and each paper has been cited at least *h* times (11). The *h*-index simultaneously measures the quantity and quality of the authors' publications (12), so it can be used to identify authors who have significant influence in the domain of pediatric MPP. We used R language's bibliometrix package to obtain the *h*-index for a total of 6,972 authors (Supplementary Table S1). Figure 4 and Table 3 display authors with high *h*-index. Susanna Esposito from the University of Milan, Italy, is the most influential author, having published 25 papers, with a total of 806 citations, and the highest *h*-index of 16. Subsequent to Susanna Esposito are Japanese scholar Kazunobu Ouchi, with 26 papers, a total of 809 citations, and the *h*-index of 15. Chinese scholar Wei Ji, Yong-Dong Yan and Italian scholar Nicola Principi tie for third place with the *h*-index of 13.

**Table 3:** Information about the top 10 authors with *h*-index.

Rank	Author	Affiliation	Total citations	*N*	*h*-index
1	Susanna Esposito	University of Milan	806	25	16
2	Kazunobu Ouchi	Kawasaki Medical School	809	26	15
3	Wei Ji	Soochow University	438	29	13
4	Nicola Principi	University of Milan	671	21	13
5	Yong-Dong Yan	Soochow University	441	33	13
6	Zheng-Rong Chen	Soochow University	413	34	12
7	Yhu-Chering Huang	Chang Gung Memorial Hospital	437	17	12
8	Matti Korppi	Tampere University and University Hospital	512	13	12
9	Naoyuki Miyashita	Kansai Medical University	470	15	12
10	Miyuki Morozumi	Keio University School of Medicine	859	16	12
11	Kimiko Ubukata	Keio University School of Medicine	859	16	12
12	Yu-Qing Wang	Soochow University	362	25	12

### 3.4. Analysis of literature co-citation

**Figure 5:**
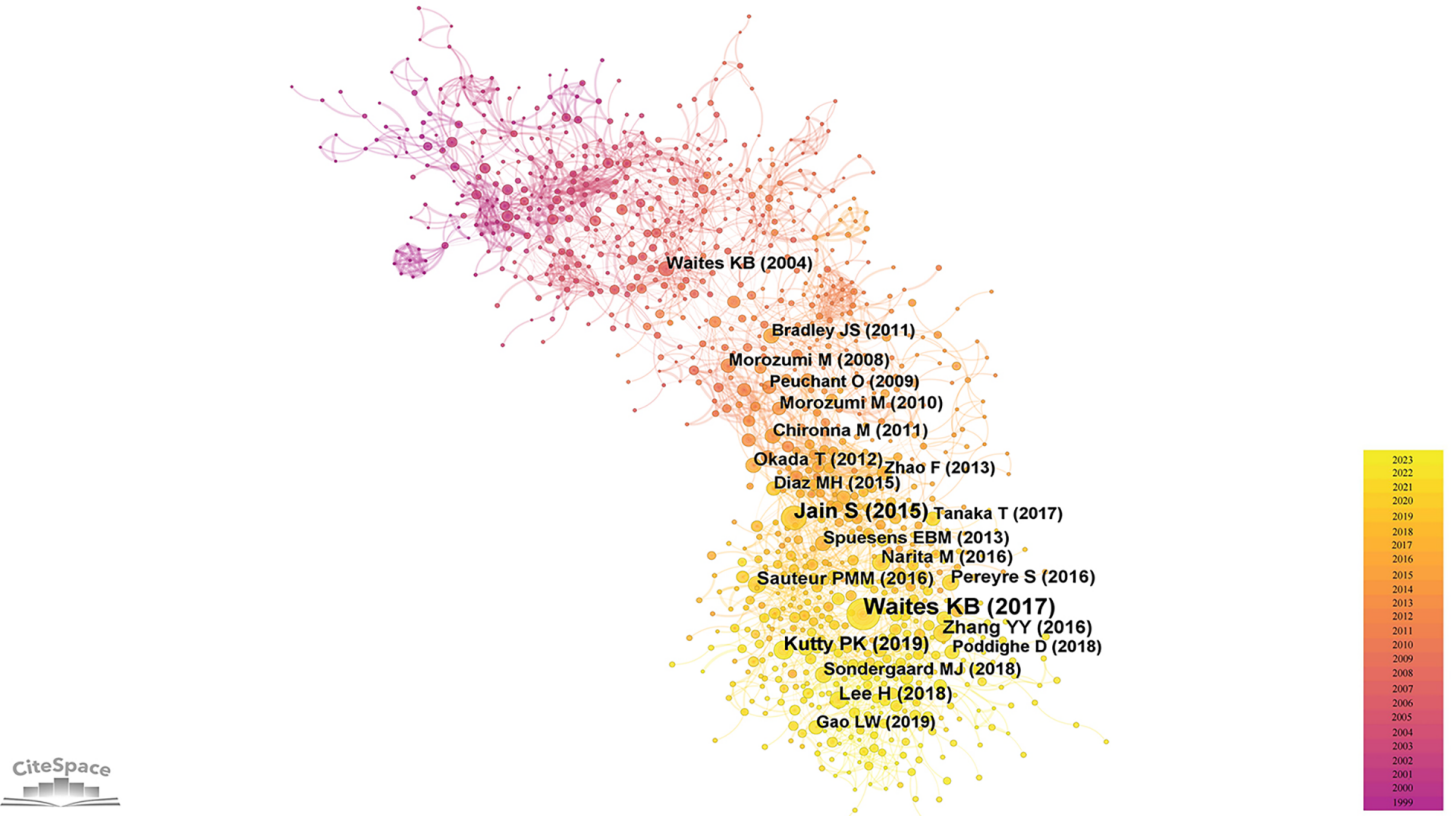
Literature co-citation network.

The foundation of a scientific field lies in its highly cited literature. In the realm of pediatric MPP, influential publications have substantially propelled the progression of research. Employing CiteSpace software for a bibliometric analysis of the cited literature, a co-citation network (Figure 5) was generated. Each node in this network symbolizes an individual reference, with the node size proportionate to its citation frequency. Since the total citations of an article is closely related to its publication time, we calculated the annual average citations for each reference (Supplementary Table S2) and listed the top ten articles in Table 4.

**Table 4:** Top 10 references with the highest number of average citations.

Rank	First author	Article	Document type	IF (2022)	Total citations	Average citations
1	Waites KB	*Mycoplasma pneumoniae* from the Respiratory Tract and Beyond. *Clin Microbiol Rev*, 2017, 30 (3):747–809. doi: 10.1128/CMR.00114-16.	Review	36.8	137	19.57
2	Kutty PK	*Mycoplasma pneumoniae* Among Children Hospitalized With Community-acquired Pneumonia. *Clin Infect Dis*, 2019, 68 (1):5–12. doi: 10.1093/cid/ciy419.	Article	11.8	57	11.40
3	Jain S	Community-Acquired Pneumonia Requiring Hospitalization among US Children. *N Engl J Med*, 2015, 372 (9):835–845. doi: 10.1056/NEJMoa1405870.	Article	158.5	91	10.11
4	Lee H	Antimicrobial therapy of macrolide-resistant *Mycoplasma pneumoniae* pneumonia in children. *Expert Rev Anti Infect Ther*, 2018, 16 (1):23–34. doi: 10.1080/14787210.2018.	Review	5.7	49	8.17
5	Tsai TA	Rational stepwise approach for *Mycoplasma pneumoniae* pneumonia in children. *J Microbiol Immunol Infect*, 2021, 54 (4):557–565. doi: 10.1016/j.jmii.2020.10.002.	Review	7.4	23	7.67
6	Gao LW	The epidemiology of paediatric *Mycoplasma pneumoniae* pneumonia in North China: 2006–2016. *Epidemiol Infect*, 2019, 147:e192. doi: 10.1017/S0950268819000839.	Article	4.2	32	6.40
7	Søndergaard MJ	Clinical manifestations in infants and children with *Mycoplasma pneumoniae* infection. *PLoS One*, 2018, 13 (4):e0195288. doi: 10.1371/journal.pone.0195288.	Article	3.7	36	6.00
8	Zhang YY	The Clinical Characteristics and Predictors of Refractory *Mycoplasma pneumoniae* Pneumonia in Children. *PLoS One*, 2016, 11 (5):e0156465. doi: 10.1371/journal.pone.	Article	3.7	47	5.88
9	Yang TI	*Mycoplasma pneumoniae* in pediatric patients: Do macrolide-resistance and/or delayed treatment matter? *J Microbiol Immunol Infect*, 2019, 52 (2):329–335. doi: 10.1016/j.jmii.2018.09.009.	Article	7.4	29	5.80
10	Chen YC	Macrolide-Resistant *Mycoplasma pneumoniae* Infections in Pediatric Community-Acquired Pneumonia. *Emerg Infect Dis*, 2020, 26 (7):1382–1391. doi: 10.3201/eid2607.200017.	Review	11.8	22	5.50

The most frequently cited article is a comprehensive review by Waites KB et al., published in *Clinical Microbiology Reviews* in 2017. This article offered an exhaustive summary of global epidemiology, pathogenic mechanisms, clinical manifestations, *in vitro* culture of MP, diagnostic techniques, antibiotic treatments, macrolide resistance, drug development against MP, and molecular typing (13). The second (14) and third (15) highly cited articles conducted multicenter, prospective clinical studies on pediatric MPP and pediatric CAP in hospitalized patients, respectively. Both studies showed that MP was a prevalent etiological agent of pediatric CAP; MPP was notably frequent in older children (≥5 years, especially between 10 and 17 years), presented usually with milder clinical symptoms; however, compared to pneumonias induced by other pathogens, MPP exhibited non-specific clinical manifestations and radiological findings. The fourth (16), ninth (17) and tenth (18) highly cited papers focused on macrolide-resistant *Mycoplasma pneumoniae* (MRMP). The fourth article reviewed the clinical features and treatment modalities of macrolide-resistant *Mycoplasma pneumoniae* pneumonia (MRMPP) in children. Based on a retrospective clinical study conducted in Taiwan, the authors of the ninth article believed that delayed treatment caused by macrolide resistance was associated with disease severity and multiple extrapulmonary complications. Similarly, through a meta-analysis, the authors of the tenth article found that patients infected with MRMP had more severe clinical manifestations and longer hospital stay than those infected with macrolide-sensitive *Mycoplasma pneumoniae*. The fifth referenced paper (19) delved into the treatment of MRMPP and refractory *Mycoplasma pneumoniae* pneumonia (RMPP) in children. The sixth (20) and seventh (21) cited article conducted clinical retrospective analyzes on the epidemiology and clinical characteristics of pediatric MPP. The eighth cited article (22) comparatively analyzed clinical data of RMPP against general MPP and concluded that elevated levels of C-reactive protein (CRP), lactic dehydrogenase (LDH), and interleukin (IL)-6 could serve as predictive indicators for RMPP.

### 3.5. Analysis of keywords

**Figure 6:**
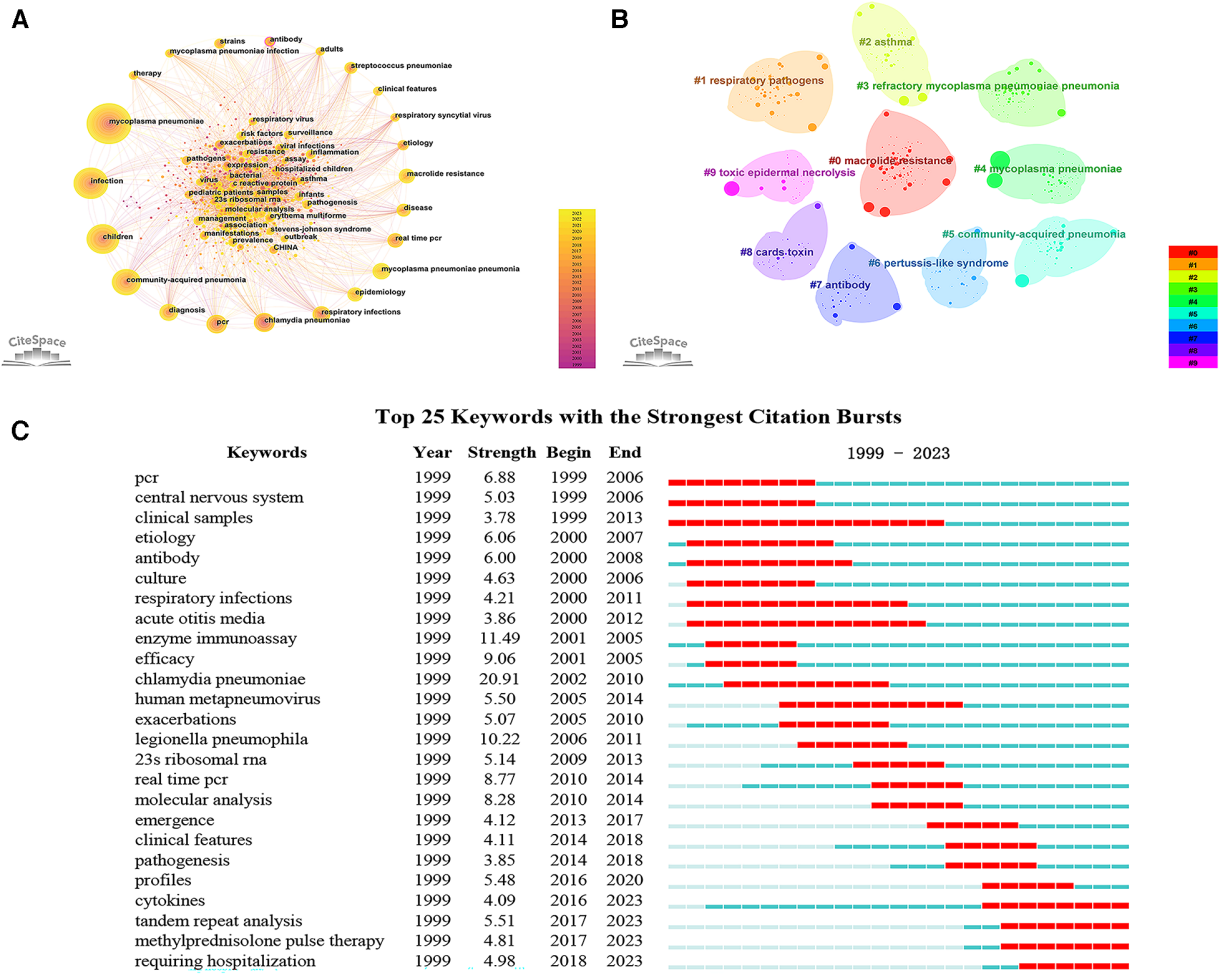
Keyword analysis. (**A**) Keyword co-occurrence network; (**B**) keyword clustering map; (**C**) top 25 keywords with the strongest citation bursts.

Keyword serves as a summarization of the research themes addressed in the academic articles. Utilizing Citespace software, keywords were extracted from a compendium of 1,729 scholarly papers, and synonymous terms were subsequently consolidated (Supplementary Table S3). A total of 636 keywords were generated. Figure 6A portrays the keyword co-occurrence network, where the node dimensions are indicative of the frequency with which each keyword appears; a higher frequency results in an enlarged node. Edges connecting these nodes represent co-occurrence within scholarly works of a thematic similarity. Table 5 delineates the 20 most frequently occurring keywords, including “MP”, “infection”, “children”, “CAP”, “respiratory infections”, and “MPP”, all of which are germane to the overarching subject matter of the present investigation. Moreover, the terms “diagnosis”, “PCR”, “real-time PCR”, “antibody”, “epidemiology”, “etiology”, “chlamydia pneumoniae”, “respiratory syncytial virus”, and “streptococcus pneumoniae” reflect an investigative focus on pathogenic diagnostics, epidemiology, and clinical characteristics. Keywords such as “macrolide resistance” and “strains” reflect the attention paid to the situation of MP resistance. Subsequent to this, a cluster analysis was employed to categorize these 636 keywords into ten clusters (Figure 6B and Table 6). Specifically, Cluster #0 is dedicated to molecular-level typing of macrolide-resistant strains; Cluster #1 investigates lower respiratory tract co-infections; Clusters #2 and #6 elucidate the interrelation of MP with other respiratory diseases; Cluster #3 delves into the biomarkers and treatment modalities of RMPP; Cluster #4 and #9 focus on extra-pulmonary complications in MPP; Cluster #5 and #7 are concerned with etiological diagnostics of MPP; and Cluster #8 pertains to pathogenic mechanisms.

**Table 5:** Top 20 high-frequency keywords.

Rank	Keyword	*N*	Rank	Keyword	*N*
1	*Mycoplasma pneumoniae*	812	11	Real time PCR	127
2	Infection	508	12	Disease	121
3	Children	499	13	Macrolide resistance	116
4	Community acquired pneumonia	395	14	Etiology	94
5	Diagnosis	239	15	Respiratory syncytial virus	85
6	PCR	233	16	Clinical features	84
7	Chlamydia pneumoniae	216	17	*Streptococcus pneumoniae*	79
8	Respiratory infections	179	18	Adults	72
9	Epidemiology	149	19	Antibody	67
10	*Mycoplasma pneumoniae* pneumonia	142	20	Strains	63

**Table 6:** Analysis of keyword cluster.

Cluster ID	Size	Label	Coverage
0	97	Macrolide resistance	Strains; tandem repeat analysis; pediatric patients; real time PCR
1	79	Respiratory pathogens	Lower respiratory tract infection; respiratory viruses; human bocavirus; influenza
2	74	Asthma	Chlamydia pneumoniae; microbiology; safety; efficacy
3	72	Refractory *Mycoplasma pneumoniae* pneumonia	*Mycoplasma pneumoniae* pneumonia; lactate dehydrogenase; bronchoalveolar lavage fluid; refractory
4	69	*Mycoplasma pneumoniae*	Encephalitis; acute disseminated encephalomyelitis; stroke; central nervous system
5	63	Community-acquired pneumonia	Etiology; pneumococcal conjugate vaccine; c reactive protein; pediatric pneumonia
6	40	Pertussis-like syndrome	Interleukin-5; montelukast; pharyngitis; asthma
7	40	Antibody	Association; pneumonia; reveals; community-acquired pneumonia
8	29	Cards toxin	Pulmonary embolism; atypical pneumonia; streptococcus pneumoniae; pleural effusion
9	26	Toxic epidermal necrolysis	Erythema multiforme; Stevens–Johnson syndrome; mucositis

**Figure 7:**
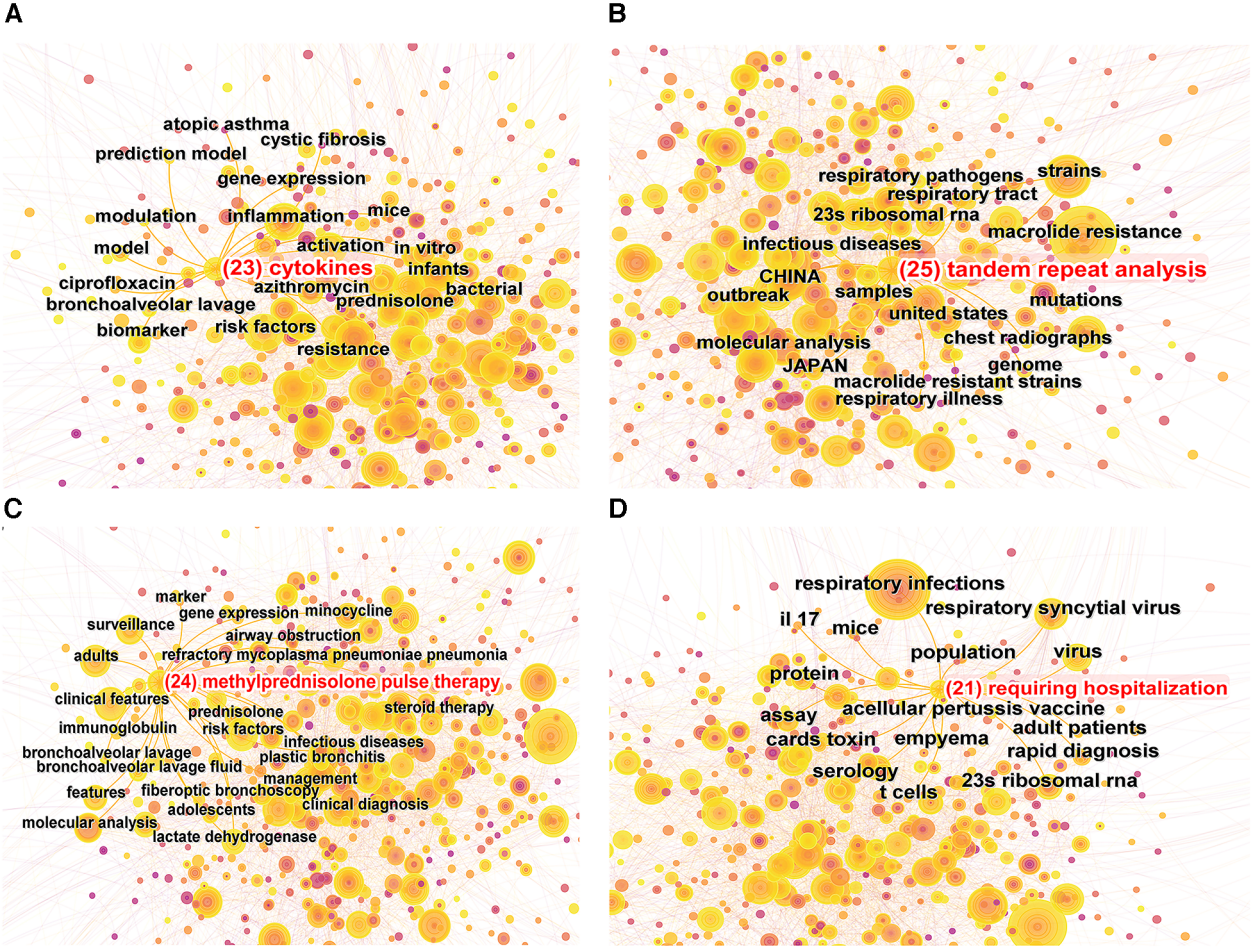
Co-occurrence map of 4 research frontier keywords. (**A**) “Cytokines” co-occurrence map; (**B**) “tandem repeat analysis” co-occurrence map; (**C**) “methylprednisolone pulse therapy” co-occurrence map; (**D**) “requiring hospitalization” co-occurrence map.

Keyword burst refers to the rapid surge in the frequency of particular keywords within a specified timeframe (8). By implementing a keyword burst analysis, one can elucidate the evolutionary trajectory of research hotspots in pediatric MPP, thereby identifying emergent research frontiers and trending avenues of inquiry. Figure 6C showcases the top 25 keywords with the strongest citation bursts from the years 1999 to 2023. The evolution of focal points in pediatric MPP research can be principally bifurcated into two phases: The initial phase spanned from 1999 to 2009, characterized by a research emphasis predominantly on MP diagnostic methodologies (PCR, clinical samples, antibody, culture, enzyme immunoassay), central nervous system damage, therapeutic efficacy, and alternative respiratory pathogens (chlamydia pneumoniae, human metapneumovirus, legionella pneumophila). The subsequent phase, commencing in 2009, manifested new hotspots including resistance mutation sites in macrolide antibiotics (23s ribosomal RNA), molecular biology assays (real-time PCR, molecular analysis, tandem repeat analysis), clinical features, pathogenic mechanisms (cytokines), and treatments (methylprednisolone pulse therapy, requiring hospitalization). Among these, the terms “cytokines”, “tandem repeat analysis”, “methylprednisolone pulse therapy”, and “requiring hospitalization” have maintained their burst status through to 2023, signifying current research hotspots and frontiers. Figure 7 depicts the co-occurrence network of four leading-edge keywords. Those tightly associated with “cytokines” primarily include “inflammation”, “activation”, “risk factors”, and “biomarker”. The term “tandem repeat analysis” is closely linked with “23s ribosomal RNA”, “strains”, “macrolide resistance” and “molecular analysis”. “Methylprednisolone pulse therapy” frequently co-occurs with “surveillance”, “adults”, “clinical features”, “risk factors” and “RMPP”. Lastly, “requiring hospitalization” is commonly co-occurring with “respiratory infections”, “virus”, “23s ribosomal RNA” and “cards toxin”.

## 4. Discussion

MPP constitutes a salient public health issue pertaining to global pediatric well-being, thus engendering an influx of research endeavors in recent years. In the current bibliometric study, we employed Citespace software, VOSviewer software and R language to methodically analyze pediatric MPP literature published in the Web of Science database, aiming to furnish researchers in the domain with a structured and visualizable knowledge framework.

### 4.1. Analysis of the number of pediatric MPP publications

Between 1999 and 2023, the publication volume concerning pediatric MPP has exhibited a steady ascendance, entering an accelerated growth phase post-2018, with an annual augmentation exceeding 100 publications. This trend underscores the burgeoning attention directed towards this disease. Concurrently, the emergence of Severe Acute Respiratory Syndrome Coronavirus 2 (SARS-CoV-2), causing Coronavirus Disease 2019 (COVID-19), has swiftly engulfed the global landscape since its initial reportage in December 2019, marking it as the most significant public health exigency of the century (23). In response, various national governments have instituted stringent containment measures, such as civilian movement restrictions, obligatory mask-wearing, and hand hygiene campaigns, thereby mitigating respiratory pathogen transmission pathways (24, 25) and inducing a palpable decrement in pediatric MPP incidence rates (26, 27). Nevertheless, our findings denoted that the years 2021 and 2022 were maximal in publication output, featuring 176 and 174 pediatric MPP-related documents respectively. The presumptive rationale may be that the pre-COVID-19 era coincided with an MPP epidemic, thereby inflating incidence rates (28, 29), which not only availed clinicians with substantial clinical data but also encouraged scholars to carry out more foundational research.

### 4.2. Analysis of the contributing nations/regions, institutions and authors of MPP in children

A total of 86 nations and regions around the world have engaged in pediatric MPP research. China and the United States emerge as the nations with the highest publication output, issuing 595 and 292 papers respectively. Cooperation between countries is relatively close, forming an intricate web of partnerships. On the contrary, as evinced by Figure 3B, the distribution of most institutions is scattered, indicating that cooperation between institutions needs to be strengthened. In terms of authorship, Italian scholar Susanna Esposito has the highest *h*-index, primarily focusing on clinical research of pediatric MPP to analyze clinical manifestations and explore the correlation between MP and asthma, upper respiratory tract infections, and extrapulmonary diseases (30–34). Japanese scholar Kazunobu Ouchi has published multiple high-quality original articles in the field of MRMP, and his *h*-index ranks second. Since 2004, Ouchi K's research team has been dedicated to conducting epidemiological investigations of MRMP infection, spotlighting molecular typing of drug-resistant strains (35–37). At the same time, he also evaluated the clinical efficacy of macrolide antibiotics, tosufloxacin, and minocycline against MRMPP in children (38, 39).

### 4.3. Analysis of research hotspots and frontiers of MPP in children

Through the statistical analysis of the references by the Citespace software, we discerned that among the ten articles with the highest average citations in this sphere, including six original research articles and four review papers. It is noteworthy that these original articles all pertain to clinical studies, signifying an exigency for substantial attention toward foundational research in the domain of pediatric MPP in the future. The principal foci of these highly-cited articles predominantly encompass the clinical characteristics of pediatric MPP, treatment modalities of MRMP, and biomarkers of RMPP.

Through the analysis of literature co-citation, as well as co-occurrence, cluster, and burst analysis of keywords, several salient issues in the pediatric MPP domain can be summarized:

#### 4.3.1. Diagnostic techniques for MP

Accurate and expeditious diagnosis of MP infection is pivotal for the initiation of appropriate antibiotic therapy (40). Current diagnostic methodologies for MP include culture, serological test, and nucleic acid amplification techniques [primarily polymerase chain reaction (PCR)], each beset with its own set of limitations (41). Traditional culture method has historically been the “gold standard” for MP diagnosis, yet this approach is hampered by the slow *in vitro* growth rate of MP and stringent environmental requirements, leading to an elevated risk of false-negative outcome (42).

Serological test, particularly complement fixation test and enzyme immunoassay, serve as pivotal tools in clinical practice for MP diagnosis (43). The surface of MP contains abundant lipid and protein antigens, which can trigger antibody responses and produce specific anti-MP immunoglobulins M (IgM) and G (IgG), becoming the main targets for serological test (44). IgM antibodies typically manifest within seven to ten days post-infection and elevated levels may persist for several months. It should be noted that infants, due to their underdeveloped immune systems, are often incapable of mounting a robust humoral immune response against MP, thus making the detection of IgM challenging (13). IgG antibodies emerge later, approximately three weeks following MP infection (45). Regardless of whether IgM or IgG is considered, relying solely on a single testing event for diagnosis is deemed unreliable; a fourfold or greater elevation of antibody levels in the convalescent phase compared to the acute phase offers diagnostic validity (46, 47).

Given the expediency, high sensitivity, and specificity of PCR techniques, they have been widely adopted for early identification of various pathogens across multiple clinical sample types, including sputum, blood, and nasopharyngeal swabs (48). Advances in molecular diagnostics have led to the diversification of PCR into conventional PCR, real-time PCR, nested PCR, and reverse transcriptase PCR among others (49). The most prevalent assay targets the P1 gene of MP utilizing real-time PCR (46). A meta-analysis by Zhang L et al. substantiated that real-time PCR offered superior performance in identifying MP infection (50). Thus, “real-time PCR” emerged as a focal term during the period of 2010–2014. Albeit numerous clinical studies affirmed higher sensitivity of PCR compared to serological test for early diagnosis in pediatric MP infections (51, 52), challenges persist due to poor cooperation among children in obtaining high-quality samples, in addition to the potential influence of PCR inhibitors and contamination, leading to false-negative or false-positive result (53). Considering the strengths and weaknesses of both serology and PCR, a combined approach was recommended for reliable diagnosis of early-stage MP infections (50).

#### 4.3.2. MRMP

Macrolides, represented by azithromycin and erythromycin, are the first-line antibiotics for the treatment of pediatric MPP (19). With the ubiquitous employment of macrolide antibiotics, resistance in MP has increasingly attracted attention, especially in East Asian countries such as Japan, China, and South Korea, where the incidence of MRMP in children exceeds 70% (35, 54, 55). The target of macrolide antibiotics is the 23S rRNA component of the MP ribosome. The ribosome serves as the nexus for protein synthesis, with 23S rRNA being an integral part of its 50S large subunit, which is further divided into six structural domains. Domain V is intricately associated with peptidyl transferase activity (56). Consequently, when mutations occur at specific sites on the domain V of the 23S rRNA in the bacterial strain, the affinity between macrolide antibiotics and MP is compromised. This impediment renders the antibiotic ineffective in inhibiting protein synthesis, thereby leading to therapeutic failure (46). Current research has identified the mutated positions as 2,062, 2,063, 2,064, 2,067, 2,611, and 2,617 (57–59). Mutations at positions 2,063 and 2,064 are the most prevalent, serving as hallmarks of macrolide resistance in MP and constituting a focal point of contemporary research (60).

Our cluster analysis of keywords indicates that research closely associated with macrolide resistance is principally oriented at the molecular typing of MP (strains; tandem repeat analysis; pediatric patients; real-time PCR). Molecular typing of MP strains aids researchers in epidemiological surveillance and in studying disease outcomes. Traditional typing method primarily targets the P1 gene through PCR-restriction fragment length polymorphism (RFLP), categorizing MP strains into types Ⅰ and Ⅱ (61). In 2009, Dégrange et al. were the first to apply multiple-locus variable-number tandem-repeat analysis (MLVA) to MP strain typing (62). MLVA includes five loci (Mpn1, Mpn13, Mpn14, Mpn15, and Mpn16); however, owing to the instability of Mpn1, contemporary analyses frequently utilize a four-loci approach (excluding Mpn1) for MP identification (63). MLVA typing exhibits higher discriminatory power compared to traditional P1 gene typing (61). Numerous studies have been explored the association between MP strain typing and macrolide resistance. Ho PL et al. found that in the Hong Kong region of China, strains MLVA 4-5-7-2 and MLVA 3-5-6-2 were predominant and were strongly associated with macrolide resistance, comprised 89.6% of cases in MRMPP patients (64). A meta-analysis by Wang et al. reached a similar conclusion, identified MLVA 4-5-7-2 as the principal type in MRMP (60). However, research by Rodman Berlot et al. failed to establish a correlation between MLVA genotypes and resistance (65), suggesting that geographic variations may contribute to disparate findings.

Given the escalating global crisis of macrolide resistance, the quest for alternative antibiotics for treating MRMP is gaining momentum. Lung DC et al. published practice recommendations for management of CAP in children, recommended the use of doxycycline for MRMP in children >8 years of age; for those ≤8 years, a cautious risk-benefit analysis should be employed, potentially resorted to doxycycline or fluoroquinolone (66). However, tetracyclines may induce enamel hypoplasia and permanent tooth discoloration, while fluoroquinolones carry the potential risk of musculoskeletal toxicity (67). Consequently, these drug classes are seldom utilized in pediatric clinical practice. In 2010, tosufloxacin was approved in Japan for treating pediatric MP infections (68). Oishi T et al. conducted resistance testing on clinical isolates of MP and found no resistance to tosufloxacin (36). Regrettably, a subsequent clinical study by the same team revealed that tosufloxacin demonstrated no significant therapeutic efficacy in treating pediatric MRMPP (69). Hence, the search for more efficacious and safer alternative treatments for MRMP remains an ongoing imperative for researchers globally.

#### 4.3.3. MP and other respiratory diseases

Clusters #2 and #6 focus on the nexus between MP and other respiratory diseases, encompassing key terms such as asthma, pertussis-like syndrome, and pharyngitis. MP infection triggers inflammatory responses in airways and augments airway resistance (70), both of which play pivotal roles in the pathogenesis of asthma (71). MP has been implicated as both an inciting and exacerbating factor in pediatric asthma, and has been confirmed by multiple studies (72–76). For instance, Biscardi S et al. discovered that in children experiencing their initial episodes of asthma, 50% exhibited acute MP infection, with MP-induced acute exacerbations of asthma constituting approximately 20% (76). A meta-analysis demonstrated a statistically significant positive association between MP infection and any type of childhood asthma. Meanwhile, some studies have also corroborated the efficacy of macrolide antibiotics in treating asthma, leading to improved pulmonary function, symptomatic relief, and a reduction in the exacerbation rate in severe neutrophilic asthma cases (77–79). However, vigilance is required concerning the risk of antibiotic resistance and the adverse effects of long-term usage; insufficient evidence exists to substantiate the use of macrolide medications in chronic or acute asthma settings (80).

Pertussis-like syndrome constitutes a clinical syndrome elicited by pathogens other than *Bordetella pertussis*, and its clinical manifestations resemble those of pertussis, commonly manifesting as paroxysmal spasmodic coughing, crowing reverberations upon cough cessation, and post-cough emesis. Infants may exhibit cyanosis, apnea, and convulsions following intense coughing episodes (81). The disease often persists for several months, with pharmacological interventions generally exhibiting suboptimal efficacy (82). MP constitutes a significant etiological factor in pediatric pertussis-like syndrome (83). Upon human entry, MP adheres prolifically to the surface of airway epithelial cells and secretes the community-acquired respiratory distress syndrome toxin (CARDS TX), which possesses ADP-ribosyltransferase (ADPRT) and vacuolating cytotoxin activities. CARDS TX is highly analogous to the S1 subunit of the pertussis toxin, which can lead to ciliary stasis, lymphocytic infiltration and increased tissue permeability, thus escalating the secretion of airway inflammatory mediators and inhibiting the clearance of mucosal secretions (84). The resultant residual secretions persistently stimulate peripheral airway nerves, thereby inducing enduring paroxysmal coughing, a mechanism akin to that of pertussis (85).

While MP is not a principal causative agent for acute pharyngitis (86, 87), a prospective cohort study indicated its association with frequent recurrences of pediatric pharyngitis (32).

#### 4.3.4. Extrapulmonary complications of MPP

MP manifests its pathological influence not only within the respiratory system but also across a multitude of organs, encompassing cutaneous, hematologic, cardiovascular, musculoskeletal, and neurological systems (88). The mechanisms underpinning these extrapulmonary manifestations can be categorized into three classes: (i) Direct: MP disseminates via the bloodstream to distal organs, instigating localized inflammatory processes. (ii) Indirect: Pathological consequences arise from immunological dysregulation incited by MP, including autoimmunity, hypersensitivity, and formation of immune complexes. (iii) Vascular occlusive: Extrathoracic pathologies ensue due to vasculitis, thrombogenesis, or a systemic hypercoagulable state induced by MP (89). Our findings indicate that neurological and dermatological connective tissue abnormalities provoked by MP attract considerable attention.

MP-associated neurological impairments commonly present as encephalitis, acute disseminated encephalomyelitis, and stroke. Based on the timing of neurological symptomatology, one can distinguish between early-onset and late-onset conditions (90). Neurological damage occurring within seven days of respiratory infection is classified as early-onset, and higher detection rates of MP in the cerebrospinal fluid of these patients have been reported (91). Narita M et al. observed a significant elevation in the levels of inflammatory markers in the cerebrospinal fluid of pediatric patients with early-onset encephalitis and aseptic meningitis (92). Consequently, some scholars posited that MP-related early-onset neurological diseases represent a form of direct extrapulmonary injury (90). The neurological symptoms following ≥8 days of respiratory infection is termed as late-onset condition, which is indirect extrapulmonary lesions with autoimmune dysregulation as the main pathological mechanism (90). Galactocerebroside C (Gal-C), a glycolipid localized within myelin of the nervous system, can cross-react with P1 adhesin and glycolipids of MP to form a Gal-C-like structure, thereby inducing cross-reactive antibody production (93).

Keywords associated with skin and connective tissue lesions induced by MP include toxic epidermal necrolysis (TEN), erythema multiforme (EM), Stevens–Johnson Syndrome (SJS), and mucositis. SJS and TEN are life-threatening dermatologic conditions characterized by extensive blistering, necrosis and desquamation of the skin (94). According to the affected body surface area (BSA), classifications include SJS (BSA <10%), SJS/TEN overlap (10% ≤BSA ≤30%), and TEN (BSA >30%) (95). SJS and TEN are predominantly triggered by adverse drug reactions or infections, with MP being the most commonly implicated infectious agent (94). EM is an acute, self-limiting dermatological disorder characterized by symmetrical targetoid erythematous lesions on the extremities, with occasional papules (96, 97). According to whether the mucosa is involved, EM can be subdivided into simple skin type and skin combined with mucosal type (98). Instances also exist where MP infection manifests solely as mucosal lesions without cutaneous involvement, previously defined variously as “atypical SJS”, “incomplete SJS”, or “EM without skin lesions” (96). However, these definitions often lead to confusion. Therefore, the American Academy of Dermatology introduced a new term in 2015, “*Mycoplasma*-induced rash and mucositis (MIRM)”, to encapsulate this condition, characterized by pronounced mucositis, absence of cutaneous lesions or manifesting only sparse vesiculobullous and/or targetoid eruptions, with a mild course and negligible mortality (99). For MP-associated SJS, TEN, and EM, the prevailing hypothesis posits that MP disseminates via the bloodstream to the skin, where it incites cytokine release and subsequent inflammatory blistering (89). The etiology of MIRM diverges; following MP infection, polyclonal B-cell proliferation ensues, leading to the production of specific antibodies and deposition of immune complexes in the skin, which subsequently activate cytotoxic T cells, resulting in mucosal injury (100).

#### 4.3.5. RMPP

If the patients with MPP still have persistent fever, unalleviated clinical symptoms and deteriorating radiographic findings after rational use of macrolide antibiotics for more than seven days, it is necessary to diagnose RMPP (101). Numerous factors contribute to the progression of MPP to RMPP, including an exaggerated host immune response, macrolide resistance, and polymicrobial infection (102). Early identification and timely therapeutic intervention are imperative for ameliorating the clinical course and prognosis of RMPP. Consequently, the identification of clinical prognostic indicators for RMPP remains a focal point of research. LDH is ubiquitously present in human tissues and serves as a frequently reported assessment metric (Cluster #3). Elevated LDH levels, released into the bloodstream due to increased cell membrane permeability during inflammation or tissue damage, offer significant diagnostic utility (103). Abundant studies corroborated that serum LDH levels were markedly elevated in RMPP and could serve as an early identification metric. However, consensus has yet to be reached regarding the predictive value of LDH in RMPP, and research findings presented some discrepancies. Zhang Y et al. conducted a retrospective analysis and ascertained that LDH ≥417 IU/L could serve as a predictive indicator for RMPP (22). Liu TY et al. determined an LDH threshold of 408 IU/L based on receiver operating characteristic curve analysis (104). Similarly, Lu A and colleagues identified an LDH level of ≥379 IU/L as indicative of a potential progression from MPP to RMPP (105). Inamura N et al. reported an LDH threshold of 410 IU/L (106). Collectively, these findings suggested an approximate LDH predictive value for RMPP around 400 IU/L. Further large-scale clinical studies are necessitated for the formulation of more precise and authoritative conclusions to guide clinical management. Additionally, other biomarkers such as CRP, erythrocyte sedimentation rate, percentage of neutrophils, 35 α-hydroxybutyrate dehydrogenase, D-dimer, procalcitonin, IL-6, IL-10, IL-18, interferon gamma (IFN-γ), and tumor necrosis factor alpha (TNF-α) have also been reported as early prognostic indicators for RMPP (22, 67, 103, 105, 107).

Regarding the therapeutic approaches for RMPP, key terms include “methylprednisolone pulse therapy” and “bronchoalveolar lavage fluid”. Severe pulmonary injury associated with RMPP is primarily attributed to an excessive host immune response rather than direct damage inflicted by MP (19). Glucocorticoids exhibit potent immunomodulatory properties (108). For RMPP, particularly severe cases, the combination of macrolide antibiotics and methylprednisolone pulse therapy is frequently utilized (109). However, a standardized glucocorticoid treatment regimen for pediatric MPP has yet to be established, and the dosage of methylprednisolone varies widely, ranging from 1 mg/kg/day to 30 mg/kg/day (102, 110). Both research groups led by You SY and Tamura A recommended a regimen of intravenous methylprednisolone at 30 mg/kg/day for three days for the treatment of severe RMPP. At the same time, they also emphasized that specific treatment dosages and duration should be adjusted according to the severity of the clinical presentation to prevent medication misuse (109, 111). The advent of bronchoscopic techniques marked a new phase in the diagnosis and treatment of RMPP. On one hand, bronchoalveolar lavage fluid serves as a lower respiratory tract sample, mitigating the interference from upper respiratory tract colonizing microbes, thereby offering compelling evidence for etiological diagnosis (112). On the other hand, therapeutic bronchoscopy can effectively remove mucus plugs, enhance pulmonary ventilation, and expedite the resolution of pulmonary inflammation (113). However, bronchoscopy is an invasive procedure; pediatricians should exercise caution in weighing the risks and benefits, generally reserving its use for cases with severe airway obstruction, such as those complicated by atelectasis or plastic bronchitis (102).

#### 4.3.6. Study on the pathogenic mechanisms

The pathogenicity of MP predominantly encompasses four aspects: direct adhesion-induced injury, exotoxin release, inflammatory damage, and immune evasion (114). In this investigation, salient keywords associated with pathogenic mechanisms include “cytokines” and “CARDS TX”, underscoring that inflammatory damage and exotoxin-mediated mechanisms are the focal points of foundational research. The MP surface harbors over 50 types of lipoproteins, the majority of which are implicated in inflammatory responses (114). These lipoproteins can be recognized by toll-like receptors (TLR1, TLR2, TLR4, and TLR6), thereby initiating the nuclear factor kappa B (NF-κB) signaling pathway and subsequently releasing a myriad of proinflammatory cytokines such as IL-6, TNF-α, and IL-1β (115, 116). These cytokines act as stimulants for human immune cells, eliciting neutrophil, lymphocyte, and macrophage infiltration into the bronchioles and alveoli, ultimately culminating in an inflammatory cascade (117). CARDS TX was initially reported in 2005 (118) and, as the sole identified exotoxin of MP, is garnering increasing attention for its role in the pathogenicity of MP (119). (i) CARDS TX can bind to human surfactant protein A, thereby promoting adhesion of MP to airway epithelial cells (120). (ii) CARDS TX, facilitated by clathrin-mediated endocytosis, gains intracellular entry and engenders the formation of perinuclear vesicles. These vesicles continually amalgamate and exert pressure on the nucleus, ultimately inducing cell death (121). The vesiculating toxicity of CARDS TX is closely aligned with the histopathological alterations in MPP, including vacuolar degeneration of ciliated cells and airway epithelial cells, leading to ciliary dysfunction and epithelial cells necrosis (119). (iii) The ADPRT activity of CARDS TX plays a pivotal role in the post-translational modification and activation of NLRP3 inflammasomes, thereby catalyzing the maturation and release of IL-1β, and triggering systemic inflammation (122). (iv) CARDS TX can induce T-cell differentiation into Th1 cells, generating IFN-γ, which in turn polarizes macrophages towards the M1 phenotype, secreting an array of chemokines. These chemokines further recruit more Th1 cells to the infection site, establishing a type Ⅰ immune response positive feedback loop and exacerbating pulmonary immune injury (123). (v) CARDS TX is implicated in the exacerbation of asthma caused by MP. Utilizing recombinant CARDS TX (rCARDS TX) to establish a murine model of airway inflammation, rCARDS TX elevated total and CARDS TX-specific serum IgE levels, triggered mast cell degranulation, and induced airway hyper-responsiveness (84, 124).

### 4.4. Strengths and limitations

To the best of our knowledge, this constitutes the inaugural work employing bibliometric methodologies to delineate research trends in pediatric MPP. Our analysis elucidates key influential nations, institutions, and authors within this domain, as well as highlights research foci. Concurrently, we identify several pressing issues warranting immediate resolution within pediatric MPP research. For instance, there currently exists no therapeutic agents that can serve as impeccable substitutes for macrolide antibiotics in the pediatric population; the relative paucity of foundational research; and with regard to the diagnosis and treatment of RMPP in children, certain aspects have yet to coalesce into unified and unequivocal standards. Certainly, some limitations are inherent to our study. Initially, this study solely incorporates English-language literature indexed in WoSCC, which may engender omissions. However, this constraint is attributed to limitations inherent in alternative databases and bibliometric analysis software. For example, citation analysis is not feasible in the PubMed database; likewise, multi-language analysis is not supported by the Citespace software. Secondly, owing to the dynamic updates of the WoSCC database, our conclusions retain their applicability solely to the current juncture. Finally, in the analysis of countries, the impact of population size on the number of publications was not taken into account. In general, countries with larger populations have more research institutions and researchers, and naturally more publications. In future research, the interference of the above factors should be eliminated as much as possible to draw more accurate conclusions.

## 5. Conclusions

In recent years, there has been a precipitous uptick in the volume of publications on pediatric MPP, indicative of heightened academic and clinical interest in this malady. China and the United States emerge as the nations with the highest publication output. Italian scholar Susanna Esposito and Japanese scholar Kazunobu Ouchi are the most influential authors. Diagnostic technologies for MP, macrolide resistance, complications associated with MPP, clinical management and diagnosis of RMPP, and underlying pathogenic mechanisms constitute the current research hotspots. Our research offers a comprehensive overview of prevailing trends in pediatric MPP studies, thereby potentially expediting the resolution of intricate challenges in clinical diagnostics and treatment.

## Supplementary material

Supplementary Table S1 The *h*-index of 6,972 authors.

Supplementary Table S2 The annual average citations for references.

Supplementary Table S3 Combination and replacement of keywords.
